# Importance of the Right Protocol in Using a Diode Laser (980 nm) for Small Oral Vascular Malformation Treatment

**DOI:** 10.7759/cureus.33643

**Published:** 2023-01-11

**Authors:** Marcia M Vidor, Luciane Hiramatsu Azevedo, Patrícia Moreira de Freitas

**Affiliations:** 1 Special Laboratory of Lasers in Dentistry, School of Dentistry, Universidade de São Paulo, São Paulo, BRA; 2 Special Laboratory of Lasers in Dentistry, Department of Operative Dentistry, School of Dentistry, Universidade de São Paulo, São Paulo, BRA; 3 Faculdade de Odontologia, Universidade de São Paulo, São Paulo, BRA

**Keywords:** diode laser, clinical case report, venous disease, oral health, vascular lesions, diode laser therapy

## Abstract

Small vascular anomalies are commonly present on the lips and other areas of the oral mucosa. These lesions are also known as venous lake lesions, and they can be treated in many ways with surgical and non-surgical methods. Laser is an arising technique for this type of procedure and its use has been growing in dental practices in the last years. The technique that uses the diode laser delivers the energy in a non-contact treatment, leading to the coagulation of the lesion in a minimally invasive manner, and for this reason, there is less chance of developing scar formation. In this report, a 56-year-old female patient had a vascular lesion on her lower lip measuring about 3.0 mm in diameter. The patient reported having it for more than 10 years. A first approach using a high-power diode laser had already been done but with no success. At this time, the diode laser was used under local anesthesia, with a flexible quartz fiber held 2 to 3 mm from the lesion, using the continuous wave mode set, 1.0 W of power for 10 seconds, with an interval of 30 seconds to prevent heat damage to the adjacent tissue. The laser was applied until the lesion appeared white and with evidence of shrinkage. In this case, two rounds of irradiation were done. After a 30-day follow-up, the lesion was repaired with no signs of scars on the lip. The diode laser is an effective technique for treating this type of lesion, with many advantages, such as providing coagulation, excellent healing, no postoperative complication, and no need for suturing. Besides all, it is also a low-cost procedure, small lesions usually require only one appointment, and this treatment is also better accepted by the patients.

## Introduction

A small oral vascular malformation is a frequent lesion usually present in older people. These lesions are also known as senile hemangioma, lip varices, and venous lake lesions. They are usually soft, and when compressed, they tend to disappear and are frequent in areas exposed to the sun. These alterations result from the growth of thin veins surrounded by fibrous tissue [1-3]. In the oral cavity, they are well-demarcated, asymptomatic, soft, purple or dark blue, usually measuring less than 10 mm, and mainly in the red lip zone and the ventral tongue surface. The formation mechanism is not well known but age is the most likely cause of these lesions since they are more frequent in people over 50 years old. These lesions lead to esthetic concerns and also doubt of malignancy, making patients seek professional advice [1,4-6].

The current treatment methods for these lesions include surgical excision, cryosurgery, electrocoagulation, photocoagulation, and sclerotherapy. But laser treatment assures an excellent treatment option and involves the less invasive method, preventing the formation of scars or discoloration in most cases. Clinically, it is a simple and fast procedure, and small lesions usually require only a single appointment. The non-contact mode using a diode laser (980 nm) is a safe and effective method for treating these lesions [5,6].

In the present clinical case, the interesting part is that previous treatment was done using this same laser and with no change in the outcome of the lesion. At the first time, an incorrect approach, such as not anesthetizing the patient, and also using lower energy and a pulse mode irradiation did not make any change to the lesion. After following the proper protocols, the lesion was treated in just one appointment. This case report highlights the importance of using a correct protocol with a 980 nm diode laser and will explain every single step used for treating this type of vascular lesion. This case report follows the CARE (Case Report) guidelines [7].

## Case presentation

A 56-year-old female patient presented with a small vascular malformation on the lower lip measuring about 3.0 mm in diameter. She informed having it for more than 10 years. After diagnosing the vascular lesion, the patient was informed of the possibility of treating this lesion with a diode laser. For this procedure, a high-power diode laser (980 nm; Thera Lase Surgery, DMC Equipment, São Carlos, Brazil) was used. The procedure was performed under local anesthesia, and the diode laser was delivered using a flexible quartz fiber kept 2 to 3 mm from the lesion, proceeding with quick circular movements. The protocol used had a mean fluency of 10J/cm2, 1.0 W of power, in a continuous wave mode, for 10 seconds of irradiation and with an interval of 30 seconds to prevent heat damage to the adjacent tissue (Table 1). The laser was applied until the lesion became white and with evidence of shrinkage. In this case, two rounds of 10 seconds each were done to achieve this result (Video 1).

**Table 1:** Clinical protocol guide

Clinical protocol
Local anesthesia using mepivacaine 2% hydrochloride epinephrine 1:100,000 (6 ml).
Laser set: 1.0 W for 10 seconds, continuous wave mode, non-contact, 2 to 3 mm distance from the lesion surface, with quick circular movement while irradiating, with 30 seconds intervals (to prevent heat damage).
Tip: Repeat the laser protocol until the lesion becomes white and with signs of shrinkage. As soon as the lesion turns white, the procedure must be stopped.

**Video 1:** Diode laser (980 nm) application Application of diode laser for treatment of the oral vascular lesion.

**Figure 1:**
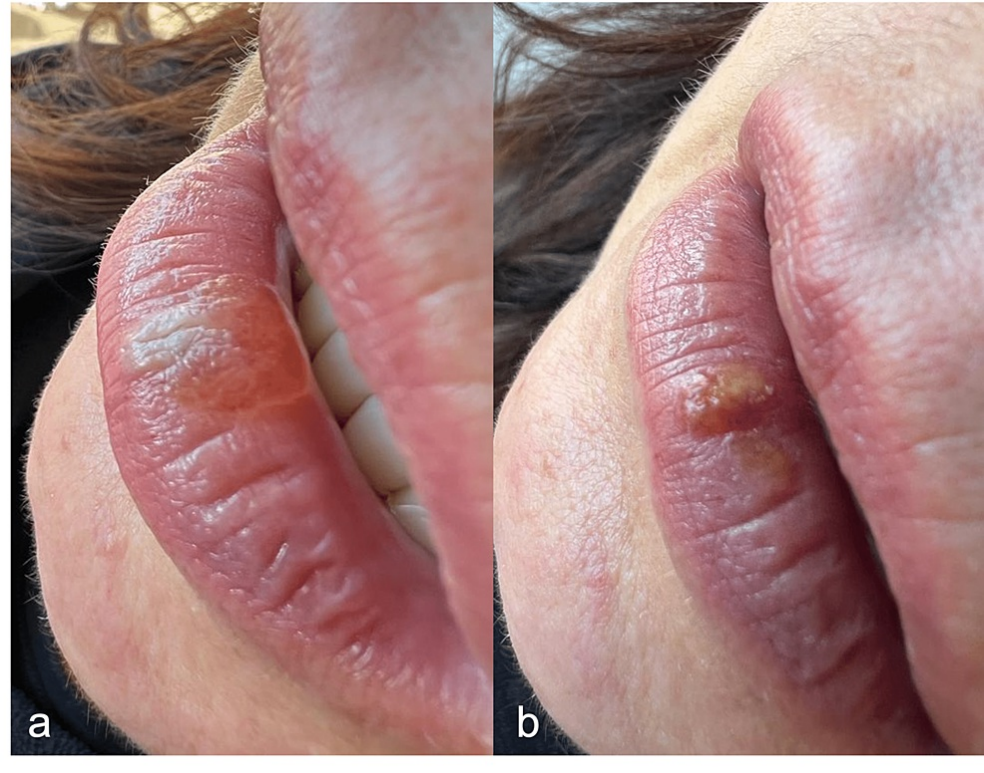
Postoperative photographs (A) 24 hours control; (B) 48 hours control.

**Figure 2:**
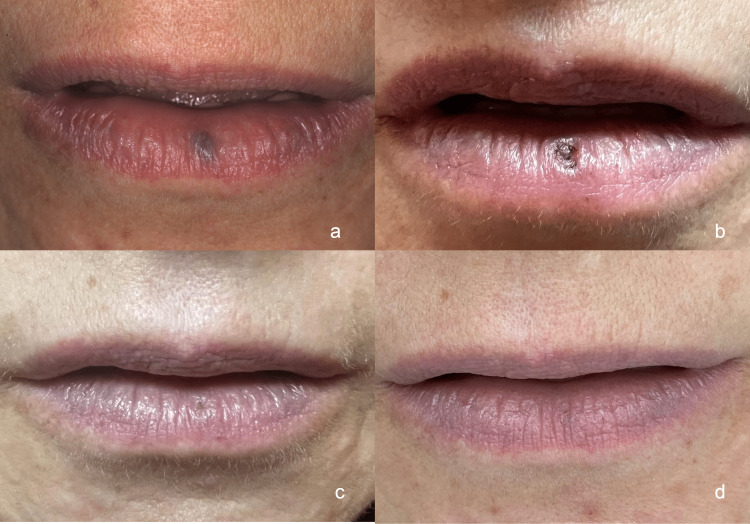
Follow-up photographs (A) Initial, (B) seven days postoperation, (C) 14 days postoperation, and (D) 30 days postoperation.

Following this single appointment of diode laser application, the patient was supervised and photographed for healing control. After 24 hours, 48 hours, and seven days of follow-up, the case evolution was carried out with photographs sent by the patient and text talk for supervision. The patient was informed and trained on how to take pictures. After 24 hours, swelling in the region where the laser was applied was observed, which was very similar to a blister. Forty-eight hours later, a reduction in the swelling was observed (Figure 1). The patient did not report any moderate or severe pain but reported only light discomfort in the first 24 hours. Seven days after the laser application, a crusted wound was observed, and after 14 days, there were barely any signs of the wound. Thirty days after the laser procedure, total healing with no signs of vascular lesions or scars on her lip was observed (Figure 2).

Patient's perspective

This patient had an esthetic concern about this lesion on the lower lip and informed us that esthetic issues were the chief complaint, and also had a concern that the lesion was becoming bigger and darker over the years. After a diagnosis of the vascular lesion, the patient was informed of the possibility to treat this lesion with a diode laser, and since it was a simple procedure, the patient decided on this technique. After the procedure, there was no report of any severe pain and only a small discomfort on immediate postoperation was noted. Fourteen days after the procedure, the patient came into the office for control, with a strong repair, and barely any signs of the vascular lesion. The patient at this point was already very happy with this repair. Thirty days postoperation, the patient came for the last control appointment and was extremely satisfied and excited with the outcome of this procedure, with no signs of vascular lesion or formation of a scar. The patient reported feeling very happy with the effectiveness and simplicity of the procedure with no discomfort.

## Discussion

Vascular anomalies are usually formed from blood vessel alterations or during endothelial proliferation and are clinically different based on their histological and histochemical aspects. They are also known as venous lakes, hemangiomas, and vascular malformations [8]. The use of a diode laser to treat these oral lesions produces fewer postoperative complications when compared to conventional techniques like scalpel surgery and also demands less operating time for the procedure. Although it might require more than one session for bigger and deeper vascular lesions [2].

The use of sclerotherapy solutions is also a choice for treating vascular lesions. Polidocanol is also one option for the treatment of these lesions to reduce their size. Besides being cost-effective, it has some complications. The polidocanol injection may cause allergic reactions, local pain, irritation, itching, and swelling [9]. Other sclerosing agents are also an alternative for treating vascular lesions such as the 3% sodium tetradecyl sulfate, which is frequently used due to its efficiency. In a study comparing this sclerosing agent with the diode laser (980 nm), it was found to have significantly fewer side effects when treating the laser group compared to the sclerotherapy group. Both treatment options were considered to be effective for treating vascular lesions, but the authors found the diode laser treatment to be a better option, especially for smaller lesions regarding less postoperative pain and fewer side effects [10].

Other studies evaluating the outcome on esthetics and recurrence of the oral venous lesions using different laser wavelengths and techniques (Nd:YAG (neodymium-doped yttrium aluminum garnet), Er,Cr:YSGG (erbium, chromium-doped yttrium, scandium, gallium, and garnet), CO2 (carbon dioxide), and diode 980 nm lasers) indicated that laser treatment of vascular lesions can be considered efficient and independent of the treatment procedure (photothermal coagulation, vaporization, or surgical excision) or the wavelength used. There was no significant difference in patient satisfaction with the esthetic outcome after six months of follow-up [5].

The non-contact irradiation of a diode laser (980 nm) is very effective when treating vascular lesions due to the high absorption by the hemoglobin at this wavelength. This process transforms the laser beam energy into heat, causing obliteration of the lesion’s blood vessels and coagulation of proteins. The application of lasers shows fewer complications for bleeding or edema, and it also causes less postoperative pain [11]. Another advantage stands for patients under anticoagulant therapy because there is no need for a drug suspension, making laser treatment even less invasive [8].

Similar results were found in another study using the diode laser for the treatment of venous lake lesions, which showed a complete healing response after a period of two to three weeks. To achieve this result, the patients needed only one session [12]. The photocoagulation technique is very effective and safe for the patient. The high-intensity laser treatment can be done using the diode or Nd:YAG, which are very appropriate for treating small or extensive lesions, but technical knowledge is very important, and the laser device should be used by an experienced trained professional [13-15]. In cases where the lesion is larger, they might require more than one laser application session [16].

Therefore, the use of a high-intensity diode laser is a very efficient option for treating these lesions and is becoming a preferred alternative for the treatment of small oral vascular lesions [6,17].

## Conclusions

The diode laser technique is a beneficial technique for small vascular malformations, with many advantages, such as providing coagulation, excellent healing, no postoperative complication, no need for suturing, and less risk for scaring. Besides all, it is also a low-cost procedure, small lesions usually require only one appointment, and this procedure is also better accepted by the patients. For all the reasons above, the diode laser is an advantageous treatment technique for these types of lesions.
